# Supplementary material for: Pharmacokinetic Study and Metabolite Identification of 1-(3′-bromophenyl)-heliamine in Rats
Source: Pharmaceuticals (Basel). 2022 Nov 29;15(12):1483. doi: 10.3390/ph15121483 (PMC9781129; doi:10.3390/ph15121483)
Supplement: Supplementary file 1 [file pharmaceuticals-15-01483-s001.zip › pharmaceuticals-2006650-supplementary.pdf]

pharmaceuticals

MDPI

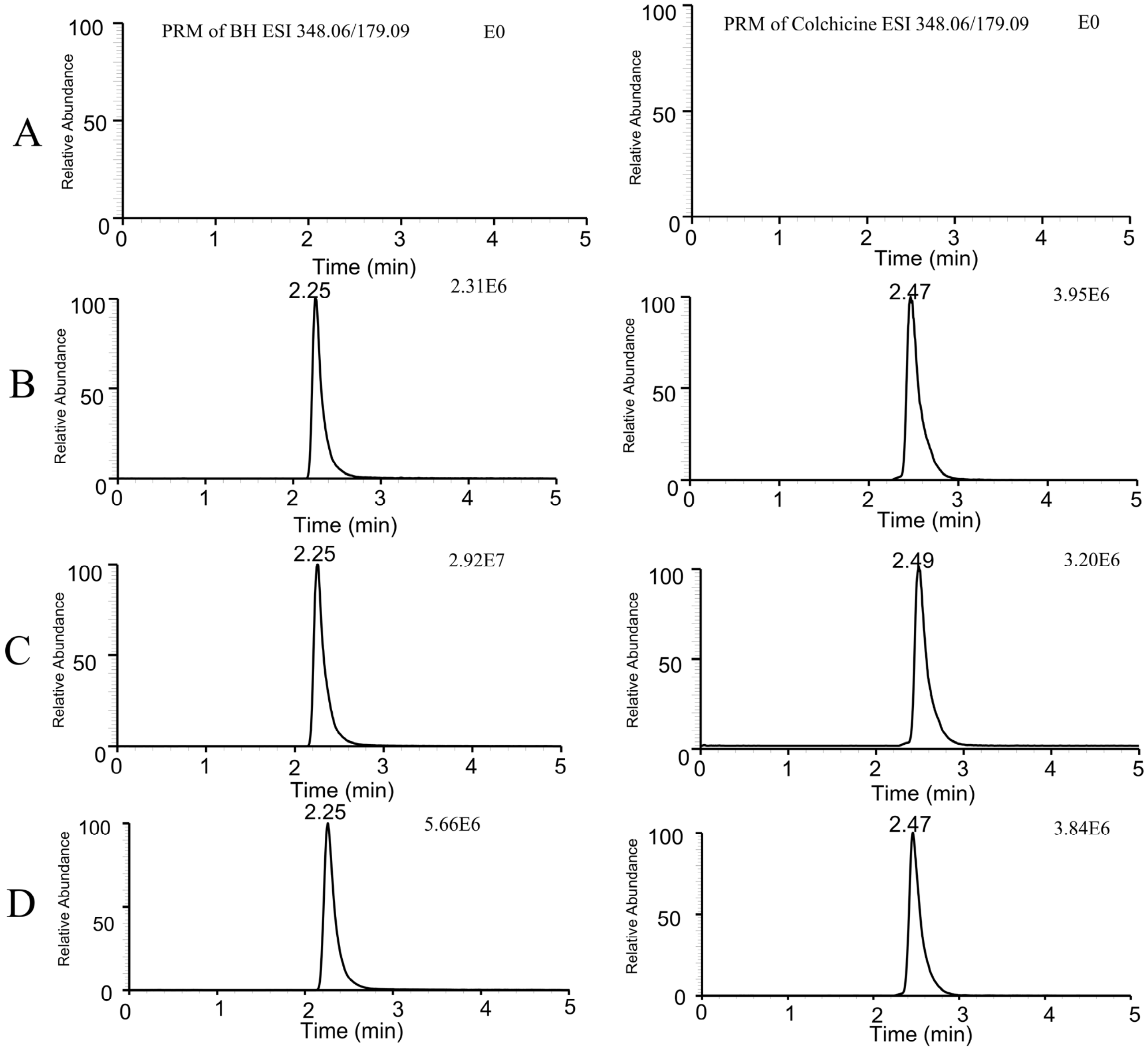

Figure S1. PRM extracted the ion chromatogram of 1–(3′–bromophenyl)–heliamine and colchicine in rat plasma: blank rat plasma (A); blank rat plasma spiked with 100 ng/mL and internal standard (B); an incurred sample after oral administration of 1–(3′–bromophenyl)–heliamine at 1.5 h (C); and an incurred sample after intravenous administration of 1–(3′–bromophenyl)–heliamine at 0.5 h (D).

Table S1. Validation parameters: intra-day and inter-day accuracies and precisions of 1–(3′–bromophenyl)–heliamine in rat plasma (n=6).

Analytes	Nominal concentration (ng/mL)	Intra-day Mean	RSD (%)	RE (%)	Inter-day Mean	RSD (%)	RE (%)
BH	1	1.02	8.53	102.00	1.03	7.33	103.00
	10	10.58	6.55	105.80	10.82	5.22	108.20
	100	99.82	4.38	99.82	96.73	3.18	96.73
	500	461.71	2.60	92.34	485.16	1.85	97.03

Table S2. The validation parameters: recovery and matrix effect of 1–(3′–bromophenyl)–heliamine and the internal standard in rat plasma.

Analytes	Nominal concentration(ng/mL)	Recovery		Matrix effect	
		Mean (%)	RSD (%)	Mean (%)	RSD (%)
BH	10	93.63	2.62	107.08	6.45
	100	97.13	3.96	106.69	8.43
	500	93.35	0.83	100.01	3.33
IS	300	101.17	3.44	108.82	4.92

Table S3. Stability results of the validation parameters under different conditions.

Analytes	Nominal concentration(ng/mL)	Accuracy (RE%)								
		4h	8h	12h	1days	7ays	15days	Stock solu-tion-15days	Room temper	Thaw
BH	10	109.51	105.98	104.12	102.64	97.41	99.05	97.05	107.36	106.02
	100	103.40	103.55	101.31	97.42	95.86	93.53	100.30	102.23	94.65
	500	98.90	96.00	98.34	97.96	97.41	93.71	99.70	98.12.	93.80
